# Use of Invasive Brain-Computer Interfaces in Pediatric Neurosurgery: Technical and Ethical Considerations

**DOI:** 10.1177/08830738231167736

**Published:** 2023-04-28

**Authors:** David Bergeron, Christian Iorio-Morin, Marco Bonizzato, Guillaume Lajoie, Nathalie Orr Gaucher, Éric Racine, Alexander G. Weil

**Affiliations:** 1 Division of Neurosurgery, 5622 Université de Montréal, Montreal, Québec, Canada; 2 Division of Neurosurgery, 7321 Université de Sherbrooke, Sherbrooke, Québec, Canada; 3 Electrical Engineering Department, 5596 Polytechnique Montréal, Montreal, Québec, Canada; 4 Neuroscience Department and Centre interdisciplinaire de recherche sur le cerveau et l’apprentissage (CIRCA), 5622 Université de Montréal, Montréal, Québec, Canada; 5 Mathematics and Statistics Department, 5622 Université de Montréal, Montreal, Québec, Canada; 6 Mila - Québec AI Institute, Montréal, Québec, Canada; 7 Department of Pediatric Emergency Medicine, CHU Sainte-Justine, Montréal, Québec, Canada; 8 Bureau de l’Éthique clinique, Faculté de médecine de l’Université de Montréal, Montreal, Québec, Canada; 9 Pragmatic Research Unit, Institute de Recherche Clinique de Montréal (IRCM), Montreal, Québec, Canada; 10 Department of Medicine and Department of Social and Preventative Medicine, 5622 Université de Montréal, Montréal, Québec, Canada; 11 Division of Neurosurgery, Department of Surgery, Centre Hospitalier Universitaire Sainte-Justine (CHUSJ), Département de Pédiatrie, 5622 Université de Montréal, Montreal, Québec, Canada; 12 Department of Neuroscience, 5622 Université de Montréal, Montréal, Québec, Canada; 13 Brain and Development Research Axis, CHU Sainte-Justine Research Center, Montréal, Québec, Canada

**Keywords:** brain-computer interface, brain-machine interface, ethics, neuroprosthesis

## Abstract

Invasive brain-computer interfaces hold promise to alleviate disabilities in individuals with neurologic injury, with fully implantable brain-computer interface systems expected to reach the clinic in the upcoming decade. Children with severe neurologic disabilities, like quadriplegic cerebral palsy or cervical spine trauma, could benefit from this technology. However, they have been excluded from clinical trials of intracortical brain-computer interface to date. In this manuscript, we discuss the ethical considerations related to the use of invasive brain-computer interface in children with severe neurologic disabilities. We first review the technical hardware and software considerations for the application of intracortical brain-computer interface in children. We then discuss ethical issues related to motor brain-computer interface use in pediatric neurosurgery. Finally, based on the input of a multidisciplinary panel of experts in fields related to brain-computer interface (functional and restorative neurosurgery, pediatric neurosurgery, mathematics and artificial intelligence research, neuroengineering, pediatric ethics, and pragmatic ethics), we then formulate initial recommendations regarding the clinical use of invasive brain-computer interfaces in children.

Brain-computer interface (BCI) is a rapidly expanding field of neuroengineering and functional neurosurgery. Brain-computer interface represents a direct communication channel between the central nervous system and a computer, bypassing primary sensory organs (ear, eyes, skin, etc) or primary effector organs (voice, arms, legs, etc). A brain-computer interface can be constituted by a neural interface allowing to extract endogenous brain signals that relate to the user's mental processes, or allowing to stimulate the brain nerve tissue in a patterned way, depending on external information collected by an artificial sensor and controller. With significant investment from both the public sector, through the European Union Human Brain Project,^1^ US Brain Research Through Advancing Innovative Neurotechnologies (BRAIN) Initiative,^2^ and US Defense Advanced Research Projects Agency (DARPA) investment in brain-computer interfaces^3^ among others, and the private sector, with companies as Synchron (Brooklyn, New York), Paradromics (Austin, Texas), and Neuralink (Austin, Texas),^4^ brain-computer interfaces are expected to reach a widespread clinical use within the next decade, helping individuals with severe neurologic disabilities and preserved cognition to connect with their environments. For instance, more than 40 patients worldwide have been implanted with temporary Utah intracortical electrodes with successful brain-computer interface control of computers, robotic arms, etc^5^; and novel fully implanted, permanent devices for long-term brain-computer interface are entering clinical^6^ or late preclinical^4^ studies. This work is closely followed by neuroethicists, who have highlighted the necessity of guidelines for neurotechnology.^7,8^ Risks of intracortical brain-computer interface extend beyond the possible complications of the neurosurgery for implantation of electrodes; as the brain-computer interface becomes an integral part of the patient, it implies important risks for the sense of identity, a risk of stigma related to having a permanently implanted device, a risk of system deficiency potentially leading to adverse outcomes, risk of brain-computer interface “hijacking” and a risk of confidentiality.^9,10^ Candidates for intracortical brain-computer interface are inherently vulnerable because of their neurologic disability, which represents an additional challenge for informed consent.^11^ Expectedly, some patients who would benefit from this technology will be children—for instance, children with traumatic cervical spinal cord injury, congenital myelopathy or myopathies, demyelinating leukodystrophies, quadriplegic cerebral palsy, or iatrogenic neurologic injury following neurosurgical interventions. Implantation of invasive brain-computer interfaces represents additional challenges in the pediatric population, notably the need for substitute consent for an investigational procedure, the capacity for abstraction and sustained attention required for the calibration and optimization of the brain-computer interface, and tailored design considerations. The existence of other, less sophisticated neuroprostheses like deep brain stimulation, vagal nerve stimulation, cochlear implants establishes a precedent on which to build for the translation of neurotechnology from investigational device, to clinical use in adults, then in children.

In this manuscript, we aim to briefly review the different types of brain-computer interfaces, then cover the technical and ethical considerations related to intracortical brain-computer interface implantation in the pediatric population. Based on a review of the literature and on the input of a multidisciplinary panel of experts in fields related to brain-computer interfaces (functional neurosurgery, pediatric neurosurgery, neuro-engineering, basic neuroscience research, artificial intelligence research, pediatric ethics), we then formulate initial recommendations regarding the clinical use of invasive brain-computer interface in children.

## Methods

First, we systematically reviewed the literature on key subjects related to the implementation of brain-computer interface protocols in children. We used a variety of keywords on 2 academic publication research engines (PubMed, Google Scholar), notably *brain-computer interface* or *brain-machine interface* combined with *intracortical*, *invasive*, *ethics*, *ethical*, *risks*, and *pediatric neurosurgery*. We retrieved manuscripts with relevant titles and abstracts and reviewed the relevant associated references. From this primary search, we identified issues frequently discussed in the ethics literature on brain-computer interface; from these topics, we generated keywords and performed a secondary targeted search, analogous to the method used in a previous scoping review.^9^

We then reviewed the past and ongoing clinical trials of brain-computer interfaces on the clinicaltrials.gov database using keywords *brain-computer interface*, *brain-machine interface*, *neuroprosthesis*, *tetraplegia*, *locked-in*, and *spinal cord injury*. We then researched the publications of principal investigators of past and ongoing trials.

Second, we built on this literature review to articulate a classification of brain-computer interface subtypes, as well as a classification of relevant technical and ethical challenges pertaining to their implementation in pediatric neurosurgery. Given that the definition of brain-computer interface is broad, we chose to focus the discussion of these challenges on motor, invasive brain-computer interfaces (see below). Invasive brain-computer interface was defined as a brain-computer interface where the electrode used to register brain signals is inside the skull.

Third, we contacted key academic actors in Quebec, with a wide variety of backgrounds related to implementation of brain-computer interface programs in pediatric neurosurgery: functional and restorative neurosurgery (C.I.-M.), pediatric neurosurgery (A.G.W.), mathematics and artificial intelligence research (G.L.), neuro-engineering (M.B.), pediatric ethics (N.O.G.), and pragmatic ethics (E.R.). This collaboration helped to critically analyze the literature and formulate recommendations related to the implementation of brain-computer interface programs in pediatric neurosurgery.

## Basic Mechanism of Brain-Computer Interface

A motor brain-computer interface uses a mathematical algorithm termed a “decoder” to estimate the user's intention, representing movement, speech, or any form of environmental interaction. The neural signal can be decoded from outside the skull (noninvasive: electroencephalography [EEG], functional near-infrared spectroscopy [fNIRS]) or inside the skull (invasive: local field potential through epidural or subdural electrocorticography, single-neuron activity [spikes] through intracortical implants; see Figure). The signal is then processed to extract informative signal components that are correlated to the mental processes of interest (eg, “move cursor up”), also called “features” (eg, an increase in activity in a subpopulation of neurons). The disadvantage of noninvasive signals like scalp EEG is that the decoded signals reflect the mean activity of a large number of neurons, with a signal that is attenuated through the thickness of the skull and the scalp, hence limiting the precision and rate of data transfer (“bit rate”) that can be achieved.^12^ Intracranial electrodes like subdural or epidural electrocorticography still record the mean activity of groups of neurons (local field potential), but in a much more precise manner, as they get much closer to the neurons they record. Finally, intracortical microelectrodes, as they penetrate the cortex to reach the cell bodies of pyramidal neurons in layer V of the cortex, can record the activity of individual neurons (action potentials, also called “spikes”).^12^ Modern intracortical brain-computer interface systems have multiscale decoders that extract information directly from binary spike events (spike, no spike) at their millisecond time scale while also adding information from continuous local field potential at their slower time scales.^5^ Basic knowledge of the anatomy and physiology of the implanted brain region helps defining a baseline interpretation of neural activity and translate it into the movement of an actuator—a prosthetic limb, for instance. Translation of brain signals into volitional movement relies heavily on signal processing and machine-learning algorithms, but also on the plasticity mechanisms in the user's brain, enabling to learn novel motor outputs. The brain-computer interface loop is closed when the user receives feedback of this action directly through their natural senses (visual feedback of the performed action) or artificially, via neural feedback (intracortical microstimulation for tactile information of a prosthetic limb, for instance).^13^ Using this feedback, the patient can learn to control the activity of this brain region to achieve more precision in controlling the actuator (computer cursor, prosthetic limb, etc). Simultaneously, engineers can refine the decoder algorithm (decoder calibration) to allow better translation of brain signals into the precise control of an actuator. This bidirectional optimization process requires many weeks of intensive training. Currently, the calibration process requires constant oversight by a team of specialized engineers and programmers, which is a major hurdle for widespread clinical use. Many factors explain this need for constant oversight: the signal features allowing to decode intent are patient-dependent, and they can change from day to day because of the submillimetric movement of the microelectrodes in relation with the neurons they record.^14-16^ Achieving automated model calibration will be a major breakpoint for the clinical translation of intracortical brain-computer interface; therefore, academic researchers and industry leaders are currently devising long-term, unsupervised recalibration algorithms of cursor brain-computer interfaces.^4,17^ The patient's learning process may also eventually be completed autonomously by the patient through an interactive app.^4^ The capacity to translate brain activity into complex, specific tasks requires the decoding of the precise activity of a large group of neurons. brain-computer interface based on noninvasive measurements such as scalp EEG or functional near-infrared spectroscopy can measure variations of activity of large groups of neurons, which can be decoded to perform simple tasks like moving a cursor; however, they lack the spatial and temporal precision to perform more complex, multidimensional tasks. Intracortical brain-computer interfaces can detect the activity of single neurons (spikes), which, if a high number of electrodes are implanted, can gather enough data to translate into more complex tasks. In a sensory brain-computer interface, sensory information is received by an artificial sensor (eg, camera and audio recorder), digitized by a computer and converted into electrical stimulation of the cortex in a specific spatial and temporal pattern, aiming to reproduce the brain's natural activity during this sensory stimulus. The goal is to evoke a conscious representation of the stimulus by the patient, as if his own sensory organs had picked up the stimulus and transmitted it to the cortex. As such, the precision of sensory brain-computer interface crucially relies on proper understanding of spatial and temporal patterns of neuronal activity during integration of various stimuli, but also the spatial specificity of the stimulating implant (area of coverage, number of electrodes, capacity for concomitant discrete stimulations) to produce properly integrated and meaningful perceptions. Some authors have used preoperative magnetic resonance imaging (MRI) magnetoencephalography (MEG) to guide the placement of electrodes and delineate areas of peak activity during imagined stimuli.^18^

### Sensory Brain-Computer Interface

Arguably, the first widely used sensory brain-computer interface introduced were *cochlear implants*—which bypass the middle ear apparatus to provide electrical input to the cochlear nerve in patients with severe hearing loss.^19^ In patients with damage beyond the cochlear nerve (like acoustic schwannoma in neurofibromatosis type 2), *auditory brainstem implants* were developed to provide electrical input directly to the brainstem cochlear nuclei using implanted electrodes.^20^ Similar implants are currently in development to restore vision by electrically stimulating the retina, optic nerve or primary visual cortex,^21^ restore smell by electrically stimulating the olfactory bulb,^22^ or restoring proprioception and tactile exploration by electrical microstimulation of the primary sensory cortex.^18^

### Motor Brain-Computer Interface

**Figure 1.**
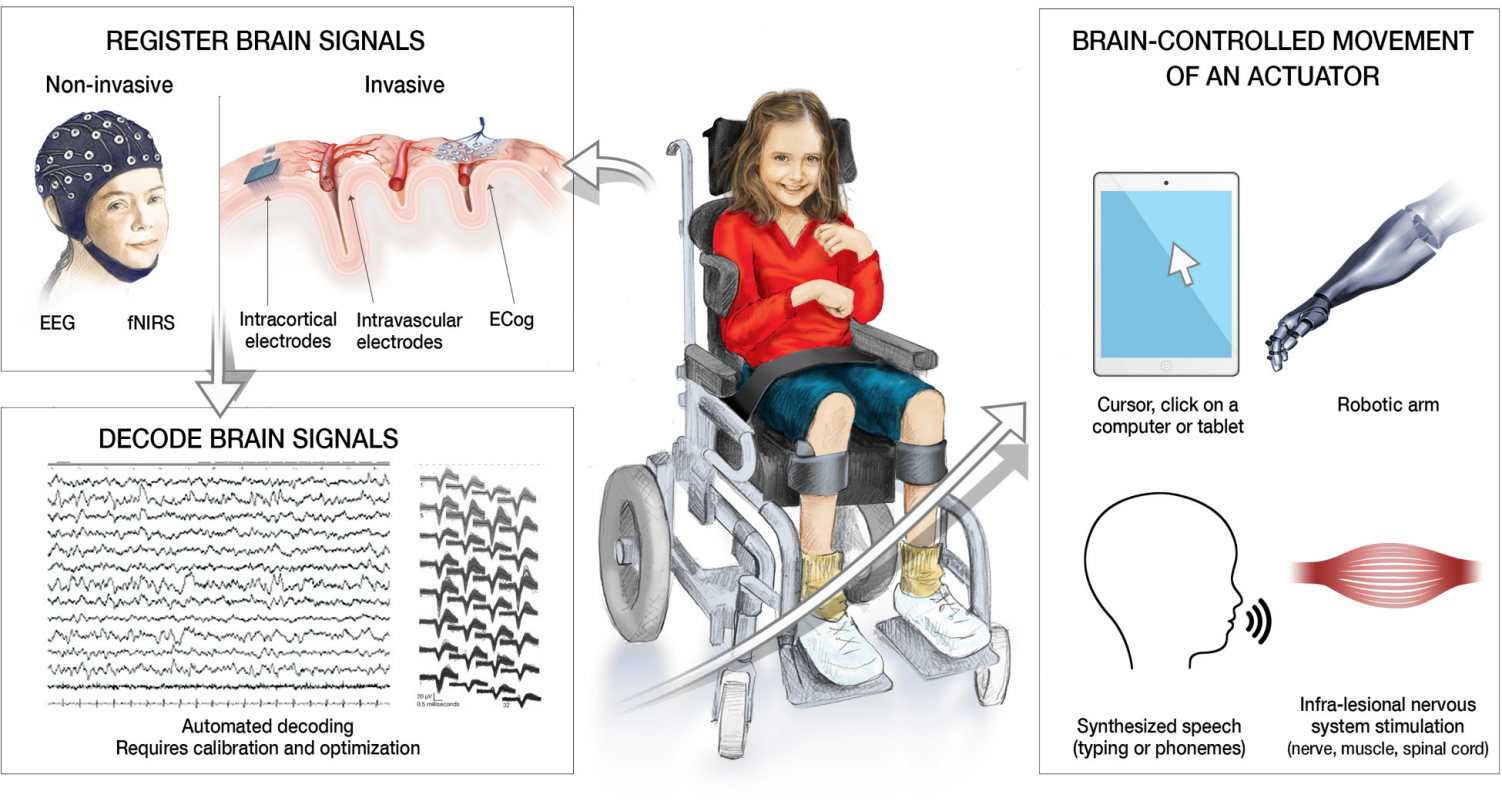
Summary of motor brain-computer interface steps and options as decoders of brain activity and actuators. Legend. Motor brain-computer interfaces (BCIs) hold promise to help children with severe neurologic disabilities, such as tetraplegia. These systems can be represented by three components.

Individuals with tetraplegia or locked-in syndrome have normal cognitive function, yet disrupted transmission of brain signals to the musculoskeletal system, resulting in severe limitations in activities of daily living. Motor brain-computer interfaces use mathematical algorithms to estimate the intended movement state from neural activity in order to control an external actuator. The decoding of brain signals can be used to control the movement of the patient's own limb through nerve^23^ or muscle^13^ stimulation, an anthropomorphic prosthetic limb,^24,25^ an exoskeleton,^26^ or a cursor on a tablet or computer.^27^ Communication can be restored through mind-controlled typing,^28-31^ and preliminary data suggest the possibility to synthesize speech at a natural rate by decoding the neuronal activity of brain regions encoding kinematic representations of articulation.^32-34^Figure 1 summarizes the putative mechanisms and actuator options of motor brain-computer interface in children.

– Register brain signals: brain signals can be decoded from outside the skull (noninvasive) or from devices implanted inside the skull (invasive). The illustration on the left shows a child with an electroencephalography (EEG) cap; functional near-infrared spectroscopy (fNIRS) is another promising technology to record brain signals noninvasively. The illustration on the right shows different variations of invasive devices to record brain signals; from left to right, intracortical electrodes (multiple small microelectrodes penetrating the cortex to record signals from layer V of the cortex), intravascular electrodes (Stentrode, placed within a cerebral sinus or vein by an interventional radiologist; records brain signals from the cortex adjacent to the vein or sinus), and electrocorticography (ECog, where a grid of electrodes is placed on the surface of the cortex to record its activity).
– Decoded brain signals: a BCI implies that the recorded cortical signals will be decoded in real time by a computer (using various machine-learning algorithms). For EEG, intravascular, and ECog signals, only the local field potentials (LFPs) can be recorded. Intracortical electrodes allow to detect “spikes” from single neurons surrounding the electrode. The left part of the illustration shows recordings from multiple intracortical electrodes, and the right part of the illustration shows the identification of individual spikes from these microelectrode recordings.
– Brain-controlled movement of an actuator: the decoding of brain signals by the computer allows to identify an intention of movement. This intention of movements translate into the movement of an actuator. Theoretically, there is an infinite number of actuators that could be controlled through the decoding of brain activity. The more practical actuators for patients with neurologic disability are represented: the control of a cursor on a tablet or computer (move cursor, click), control of a robotic arm (reach and grasp objects), control of a speech synthesizer (to either produce synthesized spoken language or written language), and infralesional nervous system stimulation (where the patient's spinal cord, nerve, and/or muscle is stimulated based on decoded intent, with either noninvasive transcutaneous systems, or invasive, surgically implanted electrodes).

### Emotional-Cognitive Brain-Computer Interface

Deep-brain stimulation has been trialed to improve symptoms of various neuropsychiatric conditions in adults, including refractory depression, obsessive-compulsive disorder, anorexia nervosa, dementia, among others.^35^ More recently, deep brain stimulation has received humanitarian device exemption for use to treat dystonia and epilepsy in children >7 years old, with some reports of its use for OCD and autism spectrum disorder with auto-aggressivity.^36-39^ When the stimulation is applied with an open-loop paradigm (with fixed parameters), deep brain stimulation represents a form of neuroprosthesis, but not a brain-computer interface, because there is no decoding of brain activity or targeted microstimulation based on sensory information. However, recent technological advancements have led to the creation of deep brain stimulation electrodes with sensing capabilities.^40^ This deep brain stimulation system has paved the way for adaptive deep brain stimulation (aDBS), which uses the recording of local field potentials to deliver personalized, data-driven deep brain stimulation treatment.^41^ Responsive Neurostimulation (Neuropace), a closed-loop neurostimulation device used to treat drug-resistant epilepsy that continuously monitors electrocorticograph activity through implanted electrodes connected to a programmer and delivers targeted neurostimulation through leads when abnormal patterns are detected.^42^ Although this device was US Food and Drug Administration approved for adult epilepsy on the basis of a large randomized controlled trial in adults,^43^ it has also been used safely and effectively in children with refractory epilepsy.^44^ Recently, the Neuropace RNS system has been successfully used to treat an individual with treatment-resistant depression.^45^ Authors have suggested closed-loop paradigms, whereby the frequency and intensity of the stimulation is modulated based on decoding of the “mood state” from neuronal activity, may improve the effectiveness of deep brain stimulation for neuropsychiatric disorders.^45,46^ In this instance, the term emotional brain-computer interface would apply because there is an automated decoding of neuronal activity that is translated into the modulation of an effector: stimulation of the brain itself. In order to work, these systems require a detailed understanding of how brain activity reflects the underlying mood of an individual at a given time.^47^

Because the definition of brain-computer interface is broad, the discussion of technical and ethical challenges related to each brain-computer interface subtype would impair our ability to provide a structured and digest analysis. We hence focused our work on the ethical challenges of implanting invasive motor brain-computer interface in children. Sensory and emotional-cognitive brain-computer interfaces evoke a distinct set of ethical considerations.^35,48^ Likewise, we will not cover eventual “neuro-augmentation” properties of intracortical brain-computer interface, which have been clearly stated as an objective for companies such as Neuralink,^4^ but are much further from clinical use.

## Brain-Computer Interface and Other Neuroprosthesis in Children—Current State

### Trials of Invasive Brain-Computer Interface Worldwide

Our clinicaltrial.gov database search found 17 studies involving intracortical motor brain-computer interface devices; of these, none involved patients younger than 18 years (see Table 1).

**Table 1.** Current clinical trials for intracortical brain-computer interfaces.

Study^a^	ClinicalTrials.gov Identifier	Country (state)	Eligible age group, y
Motor BCI			
BrainGate2: Feasibility Study of an Intracortical Neural Interface System for Persons With Tetraplegia	NCT00912041	USA (5 centers)	18-75
Brain-Machine Interface for Individuals With Tetraplegia	NCT01364480	USA (Pittsburgh)	18-70
Cortical Recording and Stimulating Array Brain-Machine Interface (CRS-BMI)	NCT01894802	USA (Pittsburgh)	22-70
ECoG Direct Brain Interface for Individuals With Upper Limb Paralysis	NCT01393444	USA (Pittsburgh)	18-70
Brain Computer Interface: Neuroprosthetic Control of a Motorized Exoskeleton (BCI)	NCT02550522	France (Grenoble)	18-55
Providing Closed Loop Cortical Control of Extracorporeal Devices to Patients With Quadriplegia	NCT01964261	USA (Caltech, University of South Carolina,)	22-65
Investigation on the Bidirectional Cortical Neuroprosthetic System (BiCNS)	NCT03161067	USA (Johns Hopkins)	22-65
An Early Feasibility Study of the ReHAB System (ReHAB)	NCT03898804	USA (Cleveland Clinic)	22-75
Restoring High Dimensional Hand Function to Persons With Chronic High Tetraplegia	NCT03482310	USA (Cleveland Clinic)	22-75
Brain Machine Interface (BMI) in Subjects Living With Quadriplegia	NCT02564419	USA (University of Miami)	22-50
Visuomotor Prosthetic for Paralysis	NCT01958086	USA (UCLA, CalTech)	22-65
ECoG BMI for Motor and Speech Control (BRAVO)	NCT03698149	USA (UCSF)	≥21
Brain-Computer Interface Implant for Severe Communication Disability	NCT04576650	USA (Johns Hopkins), Netherlands (Utrecht)	22-75
Utrecht Neural Prosthesis (UNP): A Pilot Study on Controllability of Brain Signals and Application in Locked-in Patient	NCT02224469	Netherlands (Utrecht)	18-75
STENTRODE First in Human Early Feasibility Study (SWITCH)	NCT03834857	USA (Mount Sinai, NY)	18-75
Brain Controlled Spinal Cord Stimulation in Participants With Cervical Spinal Cord Injury for Upper Limb Rehabilitation (UP2)	NCT05665998	Lausanne, Switzerland	18-75
Brain-Controlled Spinal Cord Stimulation in Patients With Spinal Cord Injury (STIMO-BSI)	NCT04632290	Lausanne, Switzerland	18-65
Sensory BCI			
Feasibility of Stimulating the Visual Cortex in Blind	NCT02747589	USA (UCLA)	18-74
A Phase I Feasibility Study of an Intracortical Visual Prosthesis (ICVP) for People With Blindness (ICVP)	NCT04634383	USA (Illinois)	18-65
Early Feasibility Study of the Orion Visual Cortical Prosthesis System	NCT03344848	USA (UCLA, Baylor, second sight)	22-74
Development of a Cortical Visual Neuroprosthesis for the Blind (CORTIVIS)	NCT02983370	Spain (Universidad Miguel Hernández de Elche)	18-70

Abbreviations: BCI, brain-computer interface; ECoG, electrocorticographic; UCLA, University of California–Los Angeles.

^a^ Not included in this table: clinical trials for auditory brainstem implants, cochlear implant or retinal implants (sensory BCI) as well as closed-loop deep brain stimulation for movement disorders, epilepsy, or other neuropsychiatric disorders

### Neuroprosthesis Trialed in Children

The only devices akin to intracortical brain-computer interface that were tested in children were auditory brainstem implants for the restoration of hearing and closed-loop neurostimulation for epilepsy. Auditory brainstem implants (ABIs) are currently indicated for children and adults with sensorineural hearing loss and irreversible damage to the cochlear nerve preventing cochlear implant; most often for infants with neurofibromatosis type 2, but increasingly used for other conditions (cochlear nerve aplasia, auditory neuropathy, etc). The optimum age for elective ABI implantation in children is between 18 and 24 months.^49^ The rationale behind an early implantation of ABI is to harness developmental plasticity to achieve better long-term auditory outcomes.^50^ This same rationale may also apply to children eligible for motor intracortical brain-computer interface, who may achieve more precise and fluid control of the actuator (cursor, prosthetic limb, speech transducer) if they are implanted at an age of greater developmental plasticity. In addition, neuromodulation devices that read brain activity are already used in children, in the form of responsive neurostimulation for refractory epilepsy (Neuropace system).^44,51^ Likewise, electrical stimulation of the brain (deep brain stimulation) is already performed for a variety of conditions like dystonia and epilepsy.^52,53^

The reasons behind the exclusion of children in trials of intracortical brain-computer interfaces are largely omitted in existing publications. There are scientific considerations, like the heterogeneity of participants in studies with low sample size, and the risk of suboptimal collaboration to the hundreds of hours of training sessions required for these studies. There are also moral considerations, as participants to these studies expose themselves to health risks with minimal improvements in their autonomy outside the laboratory setting. Indeed, these devices usually need to be plugged into the laboratory computer to work (and in most cases cannot be used at home or elsewhere) and are usually planned to be explanted after the study period. The benefits of participating in these studies currently lie in the desire to advance science, the collateral benefit of social interaction through the trial, and the hope of accessing a long-term implant after the study period.

### Noninvasive Brain-Computer Interface in Children With Severe Neurologic Disability

Although no child with neurologic disability has been implanted with an intracortical brain-computer interface, many trials of noninvasive brain-computer interfaces have been conducted in children to this date. A recent systematic review identified 12 studies of noninvasive brain-computer interface in children: 7 studies focused on brain-computer interfaces for communication and 5 on mobility, and most used EEG signal.^54^ Most trials enrolled patients with quadriplegic cerebral palsy, with severe neuromotor impairment (Gross Motor Function Classification System level V, no or minimal hand use), impaired communication (nonverbal or very limited), and relatively preserved cognitive functioning. Reactive brain-computer interface paradigms rely on event-related potentials. A popular event-related potential leveraged in brain-computer interfaces include the P300, a large positive parietotemporal deflection that occurs around 300 ms after an “oddball” visual stimulus; the steady-state visual evoked potential (SSVEP) and auditory steady-state response, wherein brain responses are evoked, respectively, by flickering lights or pure tones at specific frequencies. Active brain-computer interface paradigms elicit machine-discernible brain signals for brain-computer interface control via deliberate mental tasks such as motor imagery, which involves the mental rehearsal of a given movement, music imagery, spelling, covert speech, and pictures.^54^ Current brain-computer interface software, however, tends to focus on simple, utility-driven applications, such as spelling grids or moving a mouse cursor. Because of current hardware and software limitations, the classification accuracy ranges from 50% to 98% and drops further for children with disabilities.^54-57^ Some researchers have been able to create brain-computer interface–controlled games for children with severe neurologic disability.^58,59^ Children with such disabilities and their parents have expressed their joy in response to testing these new activities, in a context where most activities usually available to them are passive, like watching a movie.^60^ At the technological level, currently available noninvasive brain-computer interface systems can be limited by their trade-off between accuracy, speed, and degrees of freedom for selection. Researchers studying noninvasive brain-computer interface in children have noted difficulties in maintaining attention and control for extended periods due to fatigue. Another potential problem for some pediatric conditions like spastic quadriplegic cerebral palsy is that the damage is generally not focal on the corticospinal tracts; hence, additional deficits of intellectual function, motor planning, executive functions, and memory may limit the precise control of actuators using brain-computer interface. To this date, patients implanted with invasive brain-computer interface had focal damage to the corticospinal tract, either at the cervical spinal cord (traumatic SCI) or at the brainstem (brainstem strokes), with a completely intact neocortex.^34,61^ Finally, at the implementation level, the inconvenience of the setup and cleanup of the hardware associated with the technology as well as its discomfort and portability may compromise integration into daily life.^54^

Finally, it is worth noting that preliminary studies of intracortical brain-computer interface control have been conducted in children with implanted stereo-encephalography electrode (SEEG) or electrocorticography grids for presurgical evaluation of refractory epilepsy (in other words, using electrodes already implanted for another reason)—with successful decoding of the direction of arm movements during a reaching task.^62-64^

Altogether, we have shown in this section that there is currently no past or ongoing trials of invasive motor brain-computer interface in children, current experience being limited to noninvasive brain-computer interface and non–brain-computer interface neural devices. The next sections will focus on delineating the technical and ethical challenges to consider before launching trials of invasive motor brain-computer interface in children with severe neurologic disabilities.

## Technical Hardware Considerations of Intracortical Brain-Computer Interface in Children

### Surgical and Hardware Considerations

There is currently no FDA-approved fully internalized system for intracortical brain-computer interface that can be used outside the laboratory setting. The Utah NeuroPort Array—96 electrodes, extending 1.0 to 1.5 mm—is currently the only commercially available, FDA-approved microelectrode array that directly targets brain-computer interface applications. The Utah NeuroPort Array is connected with a wire to a pedestal on the patient scalp, which is connected to a computer through an external wire. A major limitation of this system is that the transcutaneous pedestal violates the barrier integrity of the skin, potentially raising the risk of infection over time. For this reason, it is FDA approved for human implantation up to 30 days, or longer with an investigational device exemption. Novel intracortical electrode arrays are currently being developed by companies such as Blackrock, Neuralink, and Paradromics, with a higher number of recording channels and fully implanted hardware with wireless transfer to the computer, designed for home use.^4^ In addition, Clinatec (CEA, Grenoble, France) has developed an implantable electrocorticographic recording device with a 64-channel epidural electrode array capable of recording electrical signals from the motor cortex for an extended period and with a high signal-to-noise ratio; this array has been implanted in a tetraplegic patient to control an exoskeleton^26^ and is being used in ongoing studies of brain-spine interface from the NeuroRestore group in Lausanne, Switzerland (NTC04632290, NCT05665998; see Table 1). A recent systematic review identified 48 adult patients implanted with the Utah array, 30 patients for less than 30 days and 18 patients for more than 30 days (up to 5 years in some patients enrolled in the BrainGate2 trial; NCT00912041). Among the 18 patients with long-term implantation (>30 days), no infection or device-related complication was reported; 1 patient had the implant removed because of skin retraction around the pedestals.^61,65^ In histologic data of animal studies, Utah arrays are known to result in reactive tissue responses including inflammation and glial and neuronal scarring near the electrode^66,67^; no data are currently available on device-related brain damage in humans implanted with a Utah array, other than the absence of clinically demonstrable neurologic deficits.^65^ The local inflammation and gliosis around electrodes can increase electrical impedance, causing devices to malfunction over time.^66^ In fully implanted intracranial neuroprosthetic systems like deep brain stimulation, a recent systematic review of more than 27 000 adult patients estimated an incidence rate of 19.04% for hardware-related complications, including intracranial hemorrhage (2.5%), infection (3.8%), lead fracture or migration (6%), extension cable malfunction (2%), skin erosion without infection (2.5%), and battery dysfunction (2%).^65^ Reported infections were predominantly found at the site of the implantable pulse generator, followed by the burr hole, and then the extension cable. In the pediatric population, some authors have reported a higher incidence of hardware-related infection—up to 10% in children who underwent deep brain stimulation for dystonia; in this study, most patients with infection (86%) had their whole deep brain stimulation hardware removed.^68^ In a review of deep brain stimulation implantation for refractory epilepsy, 4 of 40 patients (10%) had hardware-related infection, 2 had skin erosions requiring system explantation, and 1 patient had electrode breakage.^53^ Deep brain stimulation leads and batteries are sized for use in adults; hence, the risks of skin erosion and hardware fracture seem to be higher in children.^53,68-70^ This is due to the developing immune system in children (predisposing them to infection and wound healing problems) and the growth putting stress on the connectors. In children, head growth should not be an issue unless the systems are placed in very young children (less than 5 years old), which will not occur for motor brain-computer interface. Because most data gathered in systematic reviews stem from clinical trials and single-center cohorts with relatively short follow-up, they may underestimate the risk of long-term complications over decades of implantation. Finally, although the risk of infection and hardware complication is well covered in the literature, less is known about the risk of brain-computer interface systems on the children's nervous system development. Most likely, a motor brain-computer interface, if it allows increasing the child communication and interaction with the environment, should help improve his development. However, for brain-computer interface systems that involve stimulation (adaptative deep brain stimulation, responsive neurostimulation), the impact of chronic neurostimulation on plasticity, neurodevelopment, and synaptic pruning is unknown; the current assumption is that the benefit on the underlying condition outweighs potential effects on neurodevelopment, plasticity, and synaptic pruning.^36,70^ In young patients with severe disabilities due to cervical spine injury or other neurologic disorders, the risk of hardware-related complications over decades of implantation will have to be weighed against the potential clinical benefits of the device on the patients’ increased capacity for communication and autonomy.

### Long-term Stability of Decoded Brain Signals

As the field of intracortical brain-computer interface is in its infancy, we currently lack data regarding the stability of decoded brain signals over long time scales. If a teenager with cervical spine injury is implanted with an intracortical brain-computer interface, they will learn to rely on this technology in their daily life for many decades. We know from initial results of intracortical brain-computer interface implants in patients with tetraplegia,^71^ as well as previous research in nonhuman primates,^72^ that the unit recording amplitude decreases over time even in the first year after implantation. Current intracortical studies must recalibrate the mapping from neural parameters to control variables on a daily basis because of recording instability, presumably due to small movements of the electrodes relative to the surrounding brain tissue, as well as cell loss and gliosis build-up.^67,73^ With manual or automated recalibration, precise brain-computer interface control was achieved over up to 5 years in patients with tetraplegia.^71,74^ It is unknown whether signals will remain stable over decades, or if explantation and reimplantation of a new electrode array will represent a safe and efficient solution if signals become too degraded. It is also unknown whether brain maturation during the child's development would improve the precision of brain-computer interface control (through neuronal plasticity) or degrade it (excessive modification of brain activity). There is a risk that reimplanting a new microelectrode array in the same cortical region will not achieve the same performance as the previous device. We can expect a child's brain to wire efficiently around the device through increased plasticity, and struggle to adapt to a new device recording slightly different neurons. This being said, we do not have sufficient data to predict this phenomenon. Ongoing development of high-density electrocorticographic arrays (which are potentially more stable over time than intracortical electrodes ^34,75^) and new-generation intracortical electrodes^4^ represent promising alternatives to the current Utah array to improve signal stability over time. In addition, the development of small, flexible electrodes that better follow the brain's movement (such as the Neuralink approach) may reduce the friction between the implant and the brain, hence limiting the glial reaction.^76^ The use of immunomodulation or immunosuppression to reduce the glial reaction around the implant has not been explored to this date. Machine-learning algorithms are under development to help mitigate signal drift and signal loss over longer time scales.^74,77^

### Data Safety in Wireless Intracortical Brain-Computer Interface

Currently, the signals recorded by the intracortical brain-computer interface are typically transmitted to an external computer through a physical wire and connector going out through the skull and scalp. Home use of intracortical brain-computer interface will require wireless communication to an external device, which represents a risk for data safety.^78^ Several authors noted that the use of wireless communication standards exposes brain-computer interface users to risk of interference from others.^8,10,79^ Neural devices storing data on a cloud open up the theoretical possibility of individuals or organizations tracking or even manipulating an individual's mental experience.^8^ A study on the public understanding of brain-computer interface indeed revealed that privacy is a significant concern for participants.^80^ In a world in which a lot of private data about individuals’ online activity is commonly monetized, it will be important to ensure that neural data are not used by companies outside of strictly therapeutic use.^81^ Also, high security standards should be put in place to prevent intrusion or compromise by third parties.^82^ Mecacci and Haselager designed a framework to assess the practical applicability of a brain-reading technology in practical scenarios, based on 5 aspects: accuracy, reliability, informativity, concealability, and enforceability.^83^ Finally, incorporating “on-board” computations, although more power-hungry in terms of battery use, reduces the necessity for a constant connection to an external computer, hence preventing the manipulation of stimulation or decoding protocols.^84^

### Obsolescence of Cortical Implants in a Rapidly Changing Field

Brain-computer interface research is perhaps one of the most vibrant and promising fields in science and medicine. The first report of successful intracortical brain-computer interface in humans dates only to 2012, when Hochberg et al reported successful reach and grasp control of a robotic arm in 2 tetraplegic individuals implanted with a 96-channel microelectrode Utah array.^85^ Now in 2022, the brain-computer interface field benefits from billions of dollars in investments from public and private entities, with impressive progress in recent years. The implanted hardware is expected to improve drastically in the years to come. For instance, the Utah array has only 96 channels for neuronal recording, needs wire connection to an external computer for analysis and creates neuro-inflammation and gliosis that reduce the signal quality over time.^86^ Likewise, implants for deep brain stimulation only comprise a handful of channels over the implanted lead, require daylong surgery for implantation, and have limited battery life. Some new deep brain stimulation systems include the possibility to concurrently stimulate and record neuronal activity,^87^ as well as rudimentary algorithms for closed-loop deep brain stimulation.^88^ In the private field, Neuralink is currently developing a brain-computer interface system that includes 3072 electrodes distributed across 96 threads in a 2-cm chip, with automated implantation by a surgical robot^4^; Paradromics is developing an electrode array of 65 536 channels designed for high-density cortical recordings^89^; and Stentrode is developing a stent-electrode array (Stentrode; Synchron, CA) of 16 sensors recording and stimulating the cortex from the superior sagittal sinus, where it is inserted using catheter venography neurointervention.^6^ Even Neuropixel, an array developed for animal studies, outperforms currently approved technologies with 384 high-density recording channels on a 1-cm chip.^90,91^ All kinds of technologies are being developed to allow embedded spike-sorting on the implanted chip, wireless transmission of brain signals to a computer or mobile device, compact inductive chargers to recharge the battery noninvasively, etc. In other words, a currently approved electrode array implanted in a child's brain for intracortical brain-computer interface will likely be outdated by the time they reach adulthood. Overall, this rapid progress is good news for patients with neurologic disabilities and probably would not justify delaying an intervention that could improve their function and autonomy. Nevertheless, the decision to undergo surgery for an intracortical brain-computer interface system should take into consideration the need for future surgeries for reimplantation of a superior array (in the case of deteriorating signal quality), and implantable pulse generator changes and upgrades, which increase the risk of hardware infection.^65^ This consideration is especially important for children who will benefit from their implant for decades. As many companies enter the field with great investment and expectations, there is a risk that eventual bankruptcies of private companies lead to the discontinuation of tools needed to update the decoding algorithms, stimulation parameters, or the hardware itself. It should be very clear from the start, in the funding of a trial and in the patients’ consent form, who will be responsible for paying for the long-term care of the implanted patients who want to either continue to use their devices or have them removed. Meanwhile, noninvasive brain-computer interface systems, using a proxy of brain activity such as functional infrared spectroscopy (fNIRS) and electroencephalography (EEG), also benefit from massive funding, and may eventually be refined to allow a more precise control of actuators.^5^ Nevertheless, because of the distortion of the signal by the skull, noninvasive brain-computer interface will likely never reach sufficiently precise measures of brain activity to allow complex brain-computer interface applications such as high speech-rate communication and prosthetic arm control.^5^ The decision to undergo surgery for intracortical brain-computer interface should thus include a consideration of current and future noninvasive options to achieve similar outcomes.

### Conflict of Interest, Publication Bias

The field of brain-computer interface and neuroengineering is highly funded, from federal agencies and the private sector.^3,8^ For academic researchers and companies alike, there is an implicit pressure to report positive and promising outcomes and downplay potential risks or complications. Among the 48 reported patients who underwent implantation of a Utah array (over more than 5 years in some patients), there is not a single report of implant infection to this date^65^ and 1 implant had to be removed due to skin retraction around the pedestal.^65^ This result is surprising: because of the violation of the skin barrier, Utah arrays have an inherently greater risk of infection compared to fully implanted devices such as deep brain stimulation, which entails a 3.8% risk of infection and 19% lifetime risk of hardware dysfunction.^65^ This may be due to underreporting of minor infection, underreporting of major infection as a cause of device explant, or due to the use of very strict pedestal care protocols in high-resource academic settings, which would be difficult to match with a broader use of the technology. There is also an implicit pressure from researchers, publishers, and media alike to amplify the performance of brain-computer interfaces and its readiness for clinical use in terms of performance and long-term reliability. For instance, the latest communication brain-computer interfaces,^28,32,34^ although improved from earlier versions, are not sufficiently reliable and efficient to be used long-term in patients’ everyday lives. The optimism in media coverage make the technologies look more reliable and closer to large-scale clinical use than they actually are. As in deep brain stimulation, it will be the role of the neurosurgeon and medical team to assist patients and their families balancing their hopes and expectations with the limitations and risks of the technology.^92^ Furthermore, financing of invasive brain-computer interface trials should require registration to clinical trial databases and strict reporting of minor and major complications.

## Considerations Related to the Use of Machine Learning and Automated Decoding Tools in Intracortical Brain-Computer Interface

In motor intracortical brain-computer interface, brain signals are automatically decoded by artificial intelligence algorithms, and translated in the movement of an actuator. Machine learning software such as deep networks learn to decode neural data by generating complex transformations that cannot be fully understood or predicted by humans; this introduces an unknown and perhaps unaccountable process (“black box”) between a person's thoughts and the technology that is acting on their behalf.^93^ This generates multiple ethical challenges. Although currently used decoder algorithms (Kalman filters, Gaussian processes) remain interpretable, access to larger data sets will enable the use of more sophisticated and powerful machine learning approaches that will amplify the “black box” problem (Table 2).

**Table 2.** Companies Developing Devices for Invasive BCI.

Company	Device	Device description	Clinical applications	Phase of development and testing
Invasive implants designed for motor BCI				
Neuralink	N1 link	Intracortical electrode array (1024 electrodes on 32 threads) Integrated rechargeable IPG. Automated robot implantation	Short-term: control of a computer for people with tetraplegia (motor cortex decoding) Intermediate term: vision restoration, motor restoration in SCI (spinal stimulation) Long-term: cognitive augmentation	Biocompatibility studies underway (animal model: pigs) Proof-of-concept studies underway (nonhuman primates) Human trials not yet started
Synchron	Stentrode brain.io	Intravenous stent with 16 electrodes for recording and/or stimulation	Control of a computer for people with tetraplegia (motor cortex decoding)	Completed first-in-human trial with 5 patients (SWITCH trial) Ongoing further human trials
Paradromics	Connexus Direct Data Interface	Subdural high-density microelectrode grid (1600 electrodes)	Various potential applications (digital device control, assistive communication, sensory restoration, etc)	Biocompatibility studies (sheep) No human studies to this date
BlackRock Neurotech	Utah Array Neuralace	Utah array: 96-electrode intracortical array; wire connection (NeuroPort) Neuralace: ≥10 000 channels, flexible subdural array	Control of digital devices, assistive communication	Human studies (BrainGate trial) in more than 40 patients with Utah. Neuralace in preclinical development, revealed november 2022
Clinatec	Wimagine	64 channels epidural ECoG	Decoding of motor intent for control of exoskeleton or spinal stimulation	BCI demonstration in 1 patient (exosqueletton control). Brain-spine interface study underway
Other invasive implants with recording capability				
Cambridge NeuroTech	Neuropixel probe	384 intracortical microelectrodes distributed on a 10-mm shank	Animal chronic recording studies. Microelectrode recording during anesthesia for DBS or epilepsy surgery	Preclinical, animal studies. Temporary implantation during anesthesia in humans
NeuroPace	RNS System	Cortical strip leads with 4 subdural electrodes for recording, depth lead with 4 electrodes for stimulation	Responsive neurostimulation for epilepsy. Some investigational use for adaptative DBS in various neuropsychiatric conditions	FDA-approved for refractory epilepsy with a nonresectable seizure focus
Medtronic	Percept PC	DBS system, where the IPG has recording capability; with possibility for closed-loop stimulation	DBS for motor disorders (recording-informed programming; eventually automated adaptative DBS)	FDA-approved for Parkinson disease, tremor, dystonia, and epilepsy

Abbreviation: BCI, brain-computer interface; DBS, deep brain stimulation; ECoG, electrocorticographic; FDA, US Food and Drug Administration; IPG, implantable pulse generator.

### Liability and Responsibility

There is a risk that decoders make wrong predictions about the patient's intended movement, potentially leading to embarrassing or dangerous situations.^8,9,11^ This could occur because of wrong decoding of neuronal activity or to interference by electrical fields outside of the brain. In the case of a robotic arm, decoding errors could trigger unwanted movement leading to injury or material damage. In the case of a language brain-computer interface, errors in decoding could alter the meaning of sentences that the patient intended to say, leading to embarrassing situations. In sensory brain-computer interface, wrong translation of sensory stimuli in electrical neuronal stimulation may lead to patients’ wrong perceptions of the environment and possible related harm. When an involuntary act is performed because of abnormal decoding, who should be considered responsible for the harm caused? Should there be responsibility for the intermediate agent (artificial intelligence) and its designer? Unlike self-driving vehicles, brain-computer interfaces rely on the volitional control of brain activity by users, which complicates the responsibility dilemma. In young patients with developing frontal lobes, it may become difficult to distinguish intended harmful motor outputs from a software-related decoding error.

### Personhood, Integrity, and Autonomy

The presence of a machine learning process to translate the patients’ intent into an observable output may impede on the sense of autonomy and self of patients who will rely on brain-computer interface in their everyday life. For language brain-computer interface, the fidelity of the decoded phonemes to the human voice will impact the patient's sense of agency and perception of himself. To improve the typing rate of language brain-computer interface, many researchers have restricted the number of possible words and relied on the use of some kind of “predictions” or “autocorrect” based on syntactic structure.^28,34^ This may restrain the way patients express themselves, with limited access to nuanced, colorful expressions or descriptions of complex concepts. Brain-computer interface control of complex actuators like a computer or robotic arm may give an impression of “non-human cyborg” or “unnatural” communication that would risk setting apart children with intracortical brain-computer interface from their counterparts.^94^ Nevertheless, because the use of devices to extend our capabilities (smartphones, wheelchairs, etc) is commonplace and part of human nature, it is expected that an increased clinical use of intracortical brain-computer interface will eventually lead to its societal acceptance and normalization. For instance, in Canada 95% of 3 775 920 individuals living with a disability use at least 1 aid or device to assist movement, communication, learning, or daily activities of life.^95^ Furthermore, intracortical brain-computer interface enabling communication can help restore personhood and social inclusion in someone who is losing the ability to interact with their loved ones and community.^96^

On the other hand, the use of advanced technology to restore function in individuals with disabilities has been previously criticized as a form of *ableism*, that is, discrimination and social prejudice against people with disabilities; for instance, taking for granted able-bodiedness as humanity's default state, and implying the inferiority of the disabled as opposed to the non-disabled.^97,98^ Many people see their disability not as a tragic event but as an important identity or experience in their lives.^99^ A good example of this concept is the pushback against cochlear implants expressed by members the Deaf community, seeing cochlear implants as an attack on deafness as a personal and cultural identifier (as opposed to a disability), advocating against its use in children born deaf.^100^ This highlights the need to approach patients with neurologic deficits with respect and humility when it comes to suggesting surgical approaches and neurotechnology to restore function.

## Ethical Issues Specific to the Pediatric Population

### Consent, Assent, and Motivation for Surgery and Calibration Sessions

Although pediatric patients generally do not have the legal capacity to make medical decisions, their assent for brain-computer interface is essential, as the success of the procedure crucially relies on their collaboration for brain-computer interface surgery and calibration. Allowing children to be involved in their neurosurgical care is empowering and gives them both identity and agency, which is the vital first step to a successful neurosurgical intervention.^101,102^ Obtaining assent from children with severe neurologic disabilities is complicated by communication limitations.^103,104^ Nevertheless, obtaining exclusive parental substitute consent without seeking explicit assent by the child should not be considered sufficient for this type of intervention. Intracortical motor brain-computer interface calibration requires regular training sessions spanning over months—each requiring a high level of concentration and motivation. If the child does not fully cooperate during calibration sessions after implantation, the process would have put the child through surgical risks without the benefits of successfully controlling an external actuator or other targeted treatment outcomes. In that regard, pediatric brain-computer interface applications cannot be direct translations of adult studies; brain-computer interface calibration protocols may have to be significantly modified from adult studies because of the different interests, attention span, and overall functioning of children's brains.^105^ Engaging children's attention for brain-computer interface calibration may require packaging cue, stimulus, and feedback presentations within a game with rewards designed to maintain focus.^106^ Even after the calibration period, the brain-computer interface control of a prosthetic may require more cognitive planning and attention than a user can achieve on a regular basis, leading to frustration.^9,107^ There are currently few data on achieving brain-computer interface control of actuators using intracranial electrodes in children.^64^ Our center is launching a study to use signals from intracerebral electrodes implanted in children with refractory epilepsy, in order to achieve brain-computer interface control of a cursor; this will provide experience regarding the strengths and challenges for achieving brain-computer interface control in children using invasive brain signals. On the other hand, as shown in an interview study with brain-computer interface users, brain-computer interface can elicit empowerment and foster self-esteem, by contributing to medical research and progress, changing the narrative from the series of bad news and the “plateau” of readaptation to the possibilities for new achievements, and break isolation by being part of the research team.^108^

### The Risk of Scientific and Mediatic Hype for Vulnerable Patients

People with severe neurologic disabilities, who are expected to benefit the most from motor brain-computer interface are also not the most suitable research subjects.^109^ There are concerns that patients with severe neurologic disabilities (tetraplegia and locked-in syndrome, for instance) could be choosing to use brain-computer interface and participate in brain-computer interface research out of desperation or as a last resort without adequately considering the risks. We must to ensure that voluntariness is not diminished by despair, leading to inappropriate consent.^110,111^ The voluntariness of patients’ consent could also be impacted by unrealistic expectations of benefit, because of the hype and lack of nuance of the media coverage of brain-computer interface applications.^93,112^ The optimism of clinicians and researchers with a developing experience with intracortical brain-computer interface may also lead to overestimating the expected benefits in the decision process.

### Lack of Research and Regulatory Approval for the Pediatric Population

For many of the novel neuroprosthetic advances, research data and regulatory approval has come much later in children compared to adults. For instance, in epilepsy, NeuroPace RNS system received approval from the FDA for use in adults with medically refractory focal epilepsy in 2012 following a landmark trial in adult patients.^43^ Medtronic's DBS System for Epilepsy (thalamus deep brain stimulation) received approval from the FDA for use in adults with medically refractory focal epilepsy in 2017 following a landmark trial in adult patients.^113^ Although refractory epilepsy is common in children, these devices are not FDA-approved (humanitarian device exemption) to this date, and the data on their use and safety in children have lagged many years behind.^37,53,114^ This often affects whether insurance will pay for devices and procedures, and some institutions feel less comfortable with therapies that are not fully FDA-approved. This problem will be heightened for motor brain-computer interface in children, as the underlying disorders qualifying for the device (eg, tetraplegia) are less prevalent in children.^115^ As outlined in Table 1, no children have been implanted with invasive recording electrodes for motor brain-computer interface, and no ongoing clinical trial is enrolling children to this date. When expert centers start implanting children for motor brain-computer interface, it will be crucial that they do so within well-funded trials with rigorous reporting of outcomes and safety data, in order to limit the time lag between FDA approval in adults vs children.

### Barriers to Access to the Technology

It is well known that access to specialized care and advanced neurotechnology is more challenging to certain populations based on geographic location, socioeconomic status, and other factors. As an example, access to deep brain stimulation for advanced Parkinson disease in Canada widely varies from one province to another, and many demographic and socioeconomic factors seem to influence access to this procedure.^116^ Brain-computer interface, by definition, will be implanted in highly specialized tertiary care centers and will require high human and material resources. This will limit equitable access to this technology around the world, and among the residents of a given country. Thought must be given to provide equitable access to this technology once it becomes available.

### Best Interests of the Children

The “best interest of the child” standard is central to pediatric bioethics. Recent studies on the decision-making process to undergo neuroprosthetic surgery for refractory epilepsy in children have highlighted different ways to interpret the risk-to-benefit balance in parents, clinicians, and children.^117-119^ Clinicians tend to focus on the primary clinical endpoint (seizure reduction in aforementioned reviews), whereas parents tend to focus on the overall quality of life and development of their child with concerns for independence and behavior; and children put great importance on their desire to be included in decision making, their trust in the medical team, their independence, and the impact of the disease and treatments on extracurricular activities.^117-119^ Considering the current ongoing intracortical brain-computer interface trials—where the actuator can only be controlled in a lab when the wires are connected to the computer, and where the implant has to be removed after completion of the study—the risk-to-benefit ratio likely does not satisfy the best interest criteria for inclusion of children in intracortical brain-computer interface trials. The progression toward wireless implanted intracortical brain-computer interface systems for long-term home use will likely be the turning point for their use in children with severe neurologic disabilities. In the meantime, children with severe neurologic disabilities may be included in trials of noninvasive brain-computer interface systems (such as EEG-based brain-computer interface), to familiarize with the technology and concepts with minimal risks, yet less precise control of external actuators. In other words, the next steps include, first, technology refinement for fully implanted systems enabling long-term home use and, second, well-conducted controlled studies in children and adults, followed by regulatory approval for general clinical use.

## Initial Recommendations for the Clinical Use of Invasive Brain-Computer Interface in Children

Given the risks associated with invasive brain-computer interface implantation and use, as well as the vulnerability of the children who may benefit from them, a strict ethical framework is needed to guide future clinical trials of invasive brain-computer interface in pediatric neurosurgery. Based on a review of the literature and on the input of a multidisciplinary panel of experts in fields related to brain-computer interface (functional neurosurgery, pediatric neurosurgery, neuroengineering, basic neuroscience research, artificial intelligence research, and pediatric ethics), we hereby formulate initial recommendations regarding the clinical use of invasive brain-computer interface in children.

1. Invasive brain-computer interface should be considered only for children who have sufficient comprehension of the procedure to give their assent to brain-computer interface implantation and express the motivation and maturity to follow the calibration protocols.^93^
2. Invasive brain-computer interface in children will require the adaptation of decoding algorithms, calibration protocols, and selection of brain-computer interface effectors tailored for the pediatric population.
3. Invasive brain-computer interface implantation will need to be performed in highly specialized academic centers of pediatric neurosurgery, with preoperative evaluation and long-term follow-up by an interdisciplinary team including the neurosurgeon, neurologist, physiatrist, neuroengineer, physiotherapist, speech/language therapy specialists, psychologists, etc.
4. Clinical trials of invasive brain-computer interface in children should include a clear plan for the long-term care of the patient after the completion of the trial, with the removal of the intracortical array or its permanent implantation for home use.
5. If the implantation of the brain-computer interface is intended for long-term use, safeguards should be put in place to ensure the long-term care of the patient, in terms of software updates, model calibration, and hardware changes, including the financial aspects.
6. From a research perspective, conducting brain-computer interface studies with noninvasive recordings,^106^ or invasive recordings obtained for another clinical indication (like SEEG) will help build expertise for brain-computer interface use and calibration in children of different ages, on which to build once fully implantable brain-computer interface systems will be available for clinical use.
7. We recommend further research to better document the preferences of young patients and their families. Indeed, one of the lessons of the literature on various forms of neurostimulation is that the outcomes sought by clinicians may not be those valued by patients.^117,119^

## Conclusion

Invasive brain-computer interface is a rapidly developing field that holds great promise to help patients with severe motor or sensory disabilities. As these technologies approach maturity for clinical use, we highlight that its use in children will require a strict ethical framework. This work represents an initial step into developing guidelines for the clinical and research use of intracortical brain-computer interface in children.
